# Effect of prenatal and postnatal malnutrition on intellectual functioning in early school-aged children in rural western China

**DOI:** 10.1097/MD.0000000000004161

**Published:** 2016-08-07

**Authors:** Chao Li, Ni Zhu, Lingxia Zeng, Shaonong Dang, Jing Zhou, Hong Yan

**Affiliations:** a School of Public Health, Xi’an Jiaotong University Health Science Center; b Department of Health Information, Shaanxi Provincial Centre for Disease Control, Xi’an, PR China; c Nutrition and Food Safety Engineering Research Center of Shaanxi Province, Xi’an, PR China.

**Keywords:** BMI-for-age *z*-scores, early school-aged children, height-for-age *z*-scores, intellectual functioning, malnutrition, rural China, weight-for-age *z*-scores

## Abstract

The aim of this study was to evaluate the effect of prenatal and postnatal malnutrition on the intellectual functioning of early school-aged children. We followed the offspring of women who had participated in a trial of prenatal supplementation with different combinations of micronutrients and who remained resident in the study field. We measured their intellectual functioning using the Wechsler intelligence scale for children (WISC-IV). Height-for-age, weight-for-age, and body mass index (BMI)-for-age were used as anthropometric nutritional status indices. Four of the 5 composite scores derived from the WISC-IV, except for working memory index (WMI), were significantly lower in low birth weight children after adjusting for confounds. All 5 composite scores, including full-scale intelligence quotient (FSIQ), verbal comprehension index (VCI), WMI, perceptual reasoning index (PRI), and processing speed index (PSI) were significant lower in stunted and underweight children. The differences in the means of WISC-IV test scores were greatest between stunted and nonstunted children. The means for FSIQ, VCI, WMI, PRI, and PSI were as follows: 5.88 (95% confidence interval [CI]: 2.84–8.92), 5.08 (95% CI: 1.12–8.41), 4.71 (95% CI: 1.78–7.66), 6.13 (95% CI: 2.83–9.44), and 5.81 (95% CI: 2.61–9.00). These means were lower in stunted children after adjusting for confounds. Our results suggest the important influences of low birth weight and postnatal malnutrition (stunting, low body weight) on intellectual functioning in early school-aged children.

## 1 Introduction

Maternal and child malnutrition remain pervasive and damaging conditions in low-income and middle-income countries.^[1]^ Malnutrition alters intelligence by interfering with the child's energy level, rate of motor development, and overall health. Poverty and low educational levels of mothers exacerbate these negative effects.^[2,3]^ Childhood intelligence quotient (IQ) is important for a successful life, and findings from a series of population-based prospective cohort studies indicate that childhood IQ is associated with further leadership success and school achievement.^[4]^ Many studies proved that improving the nutrition of school-aged children can contribute to their school performance and educational achievement and, thereby, to the long-term socioeconomic development of individual and country.^[5]^ Despite advocacy for nutrition intervention for women during pregnancy and children in early school age, there is lack of data on nutritional indicators and on cognitive development for this age in most developing countries, including rural China.^[5,6]^

Nutritional status in utero, for which birth weight is often used as an indicator,^[7]^ and for children above 5 years of age, stunting, thinness, and low body weights are the main indicators used to measure the nutritional status of individual children.^[8]^ Among these indicators, stunting and thinness are used as nutritional indices for cumulative and acute malnutrition, respectively. Low body weight is a composite indicator; it can reflect “thinness”, indicating acute weight loss, “stunting”, or both.^[9]^

Lynn^[10]^ noted that the average scores on intelligence tests increased with improvements in prenatal and early postnatal nutrition. This result reflected the important role of prenatal and postnatal nutrition on intellectual development. In addition, many recent studies have suggested that there is a negative association between low birth weight and lower school-aged IQ scores.^[11]^ Similar associations between stunting and childhood intelligence have also been found in many other studies. The results of previous studies were consistent regarding the effect of prenatal and postnatal malnutrition. However, associations between low body weight and poor cognition or school achievement are less often available than those for stunting,^[12,13]^ and there was lack of data on estimating the effect of prenatal and postnatal malnutrition on overall cognitive ability and different dimension of intellectual development.

Therefore, in this paper, we used the data from a cohort study to estimate the effect of prenatal and postnatal malnutrition on intellectual functioning in early school-aged children whose mothers received daily prenatal micronutrient supplements in a controlled, cluster-randomized, double-blind trial conducted between 2002 and 2006.

## 2 Methods

### 2.1 Setting

The present study was conducted in Changwu and Bing counties, which are 2 rural counties situated in the western part of Shaanxi Province. The areas of the 2 counties are approximately 567 square kilometers, with 9 townships and 160 villages in Changwu County, and 1202 square kilometers with 13 townships and 247 villages in Bing County. In 2007, the total population of these 2 poor rural counties was approximately 497 thousand, of which 90.3% were engaged in agriculture.

### 2.2 Study design and participants

The participants in the present study were from a large trial conducted from 2002 to 2006 that aimed at determining the effects of micronutrient supplementation during pregnancy on birth weights. The details of the large trial have been described elsewhere.^[14,15]^ Briefly, this double-blinded, cluster-randomized, and controlled trial was conducted in 2 rural counties. We used computer-generated random numbers stratified according to county and township and randomly assigned villages to the 3 supplementation groups (daily folic acid, folic acid plus iron, or multimicronutrient supplements) before recruitment. Pregnant women in the same village received the same supplement tablets daily from enrollment until delivery. A total of 107 participants were excluded because of gestational age ≤28 weeks (n = 79), serious illness that included the mental handicap of the parents (n = 12), and abnormal reproductive histories (n = 16).

Households with eligible children were invited to participate in the present follow-up study, and the interviews were administered at the local school or hospital in a standardized manner. We followed the offspring of women who had participated in the trial of prenatal micronutrient supplementation and who remained resident in the study area from October 2012 to September 2013. In the present study, we excluded children with birth defects (n = 78) and children who had a fever when the study was conducted. However, no child was lost from the study due to fever because we were ultimately able to schedule another time to interview after the child had recovered. The examiners were trained rigorously and have demonstrated high levels of consistency with each other, including for psychological tests, anthropometric measurements for children, and the collection of household information for parents.

### 2.3 Anthropometry and malnutrition definition

The data for birth weight was reliable because it was measured with an electronic scale with precision to the nearest 10 g within 1 hour of delivery by hospital nursing staff trained to measure birth weight. Height (barefooted) was recorded to the nearest 0.1 cm using a calibrated stadiometer (Model SZG-210, Shanghai JWFU Medical Apparatus Factory, Shanghai, China), and weight was recorded to the nearest 0.1 kg in standard school clothing (without shoes) using an electronic scale (Tanita BC-420, Tanita Corporation, Tokyo, Japan). Weight-for-age *z*-scores (WAZ), height-for-age *z*-scores (HAZ), and body mass index-for-age *z*-scores (BAZ) were derived from these observational data based upon 2007 references.^[16]^ Low body weight, stunting, and thinness were defined as WAZ, HAZ, and BAZ scores of ≤2, respectively.

A face-to-face interview was conducted in households using a household questionnaire. Written informed consent was obtained from all participants. Socioeconomic background factors such as the number of older siblings, educational levels, and occupations of parents were collected from the interview. The anthropometric measurements were collected by trained staff using standard procedures. The study was approved by the Human Research Ethics Committee of the Xi’an Jiaotong University Health Science Center.

### 2.4 Psychological testing

Wechsler tests are among the most widely used intelligence tests in the world. The fourth edition and also the latest edition of Wechsler intelligence scale for children (WISC-IV) which can be applied to children aged 6 to 16 years was used to assess the cognitive ability of children in the present study. The WISC-IV consists of 10 core subtests and 4 supplemental subtests. The core subtests are Block Design, Similarities, Comprehension, Vocabulary, Picture Concepts, Digit Span, Letter–Number Sequence, Matrix Reasoning, Coding, and Symbol Search, and supplemental subtests are Picture Completion, Information, Cancellation, and Arithmetic. In addition, the WISC-IV generates a full-scale intelligence quotient (FSIQ), which represents overall cognitive ability, and 4 other composite scores (processing speed index [PSI], verbal comprehension index [VCI], working memory index [WMI], and perceptual reasoning index [PRI]), which represent different domains of cognitive function.^[17]^

Currently, the WISC-IV has been commercialized in China, and Chinese norms have been established. China has standardized the WISC-IV to be culturally appropriate. The reliability and validity of these norms have also been measured and shown to be satisfactory.^[18]^

### 2.5 Statistical analyses

All data were double-entered into the data management system for verification and checked manually for completeness. Extremum, range, and logical checks were conducted for accuracy.

The primary analyses were based on an intention to treat. Statistical significance was set at a *P* value <0.05 for all statistical tests, and testing was two-sided. The distributions of household demographics and socioeconomic status in study fields were described by their means, standard deviations, and percentages. A wealth index was constructed from 16 different household facilities or assets with a principal component analysis method,^[19]^ and wealth index was commonly categorized into thirds as an indicator of the richest, middle income, and poorest households. HAZ, WAZ, and BAZ scores were calculated with the use of the WHO Anthro Plus Software package, which applies the 2007 WHO reference values.^[16]^ In the present study, multilevel analyses with township to level 3, village to level 2, and individual to level 1 were used to compare the FSIQ, VCI, WMI, PRI, and PSI. Estimations of the coefficients and 95% confidence intervals (CIs) were made.

Previous studies indentified poverty, malnutrition, and social factors were detrimentally affect cognitive development of children.^[19–21]^ In addition, the effect of prenatal iron supplementation on intellectual functioning in early school-aged children was also reported in previous study.^[22]^ Therefore, we considered the following variables as potential confounders in the multivariate adjusted analysis: socioeconomic background (age of children, maternal age, sex of children, educational level of parents, occupation of parents, household wealth index, older siblings of children, and school of children), type of prenatal micronutrient supplementation, and gestational weeks at birth.

To test the reliability of the analysis, we repeated the analyses after adding birth weight, HAZ, WAZ, and BAZ scores as continuous variables into the multilevel model. Data were analyzed using the STATA software, version 12.0 (Stata Corp LP, College Station,Texas 77845, USA).

## 3 Results

By September 2013, 1643 of the participants had moved out of the study area and 96 had died. Of the remaining 2865 children eligible for inclusion in present study, we followed 1744 of them during the study period.

Table 1 shows the baseline characteristics of the investigated households and their children. The mean age of the children was 8.78, and more boys were followed up in 2 counties. The prevalences of low birth weight, stunting, low body weight, and thinness in the investigated children were 4.6%, 3.7%, 6.2%, and 6.5%, respectively. For WISC-IV test scores of the children, the mean of FSIQ was 89.48 (Standard deviation [SD] 13.39), and the means of 4 other composite scores (VCI, WMI, PRI, and PSI) were 87.83 (SD 15.90), 91.04 (SD 12.34), 93.12 (SD 13.88), and 95.68 (SD 13.34), respectively. These means were lower than for children in urban China (means of FSIQ, VCI, WMI, PRI, and PSI = 100). In addition, the distribution of baseline characteristics, nutritional status, and intellectual functioning of children in the 2 counties were not balanced. The socioeconomic status, nutritional status, and intellectual functioning of children in investigated households were better in Changwu County. Obviously, intellectual functioning was related to the better socioeconomic status of Changwu County.

**Table 1:**
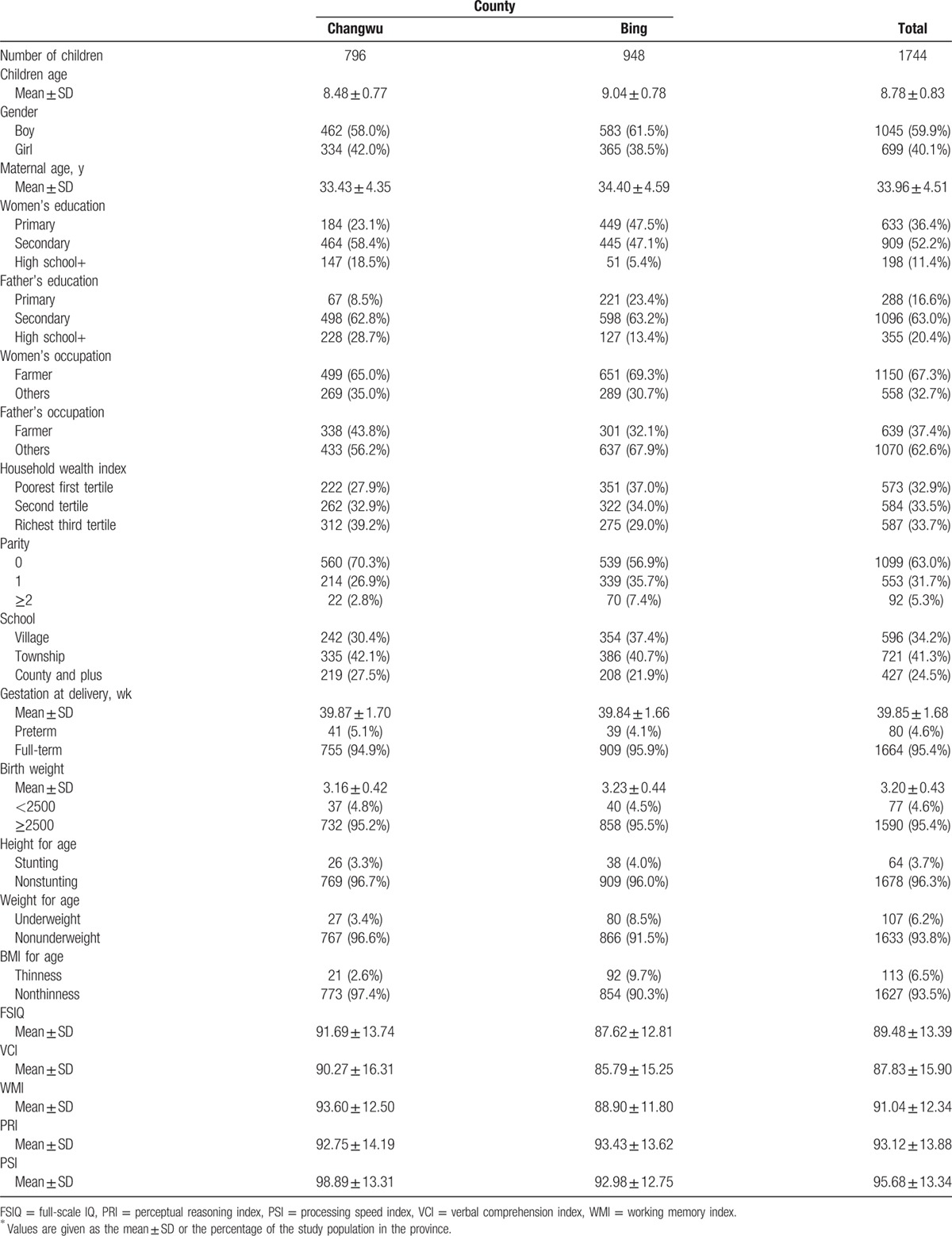
Characteristics of study population by study fields^∗^.

Table 2 shows the means of the WISC-IV test scores of children in different malnutrition status groups. The WISC-IV test scores of children were lower if they were of low birth weight, stunted, underweight, and thin. The greatest mean differences for FSIQ, VCI, WMI, PRI, and PSI were between the stunted and nonstunted groups. The smallest mean differences were between the thin and nonthin groups.

**Table 2:**
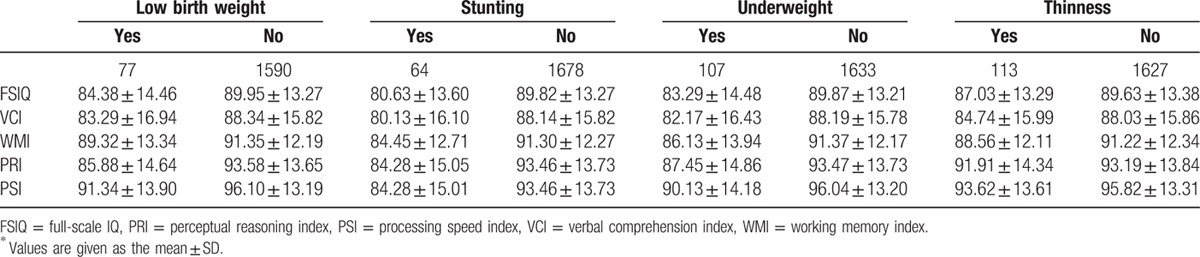
Mean scores on WISC-IV tests by birth weight, stunting, and wasting in children^∗^.

As shown in Table 3, low birth weight was significantly associated with lower WISC-IV test scores. Before adjustment, the means of FSIQ, VCI, WMI, PRI, and PSI were 5.49 (95% CI: 2.59–8.38), 5.08 (95% CI: 1.64–8.51), 2.00 (95% CI: 0.70–4.70), 7.66 (95% CI: 4.62–10.69), and 4.79 (95% CI: 1.86–7.72), respectively, and were lower in low-birth-weight children. After adjusting for confounds, these mean differences between low birth weight and normal birth weight were slightly attenuated and the association was no longer significant between low birth weight 1.64 (95% CI: −1.11 to 4.39) and WMI.

**Table 3:**
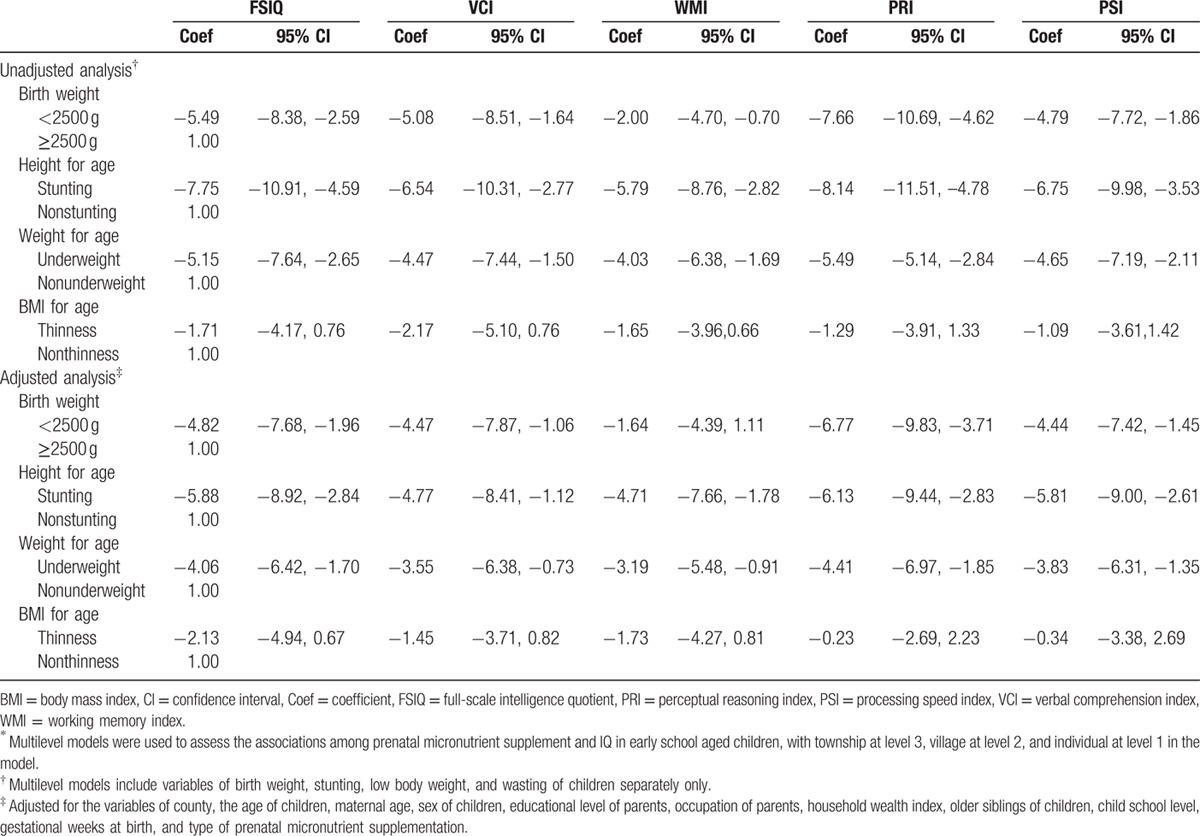
Results of multilevel models into the relationships between birth weight, stunting, low body weight, wasting, and WISC-IV scores of children^∗^.

Stunting was strongly and negatively associated with WISC-IV test scores before and after adjustment. The means of FSIQ, VCI, WMI, PRI, and PSI were 5.88 (95% CI: 2.84–8.92), 5.08 (95% CI: 1.12–8.41), 4.71 (95% CI: 1.78–7.66), 6.13 (95% CI: 2.83–9.44), and 5.81 (95% CI: 2.61–9.00), respectively, and were lower in stunted children after adjusting for confounders. The association was robust when adjusted for potential confounders. Differences in WISC-IV test scores were significant between underweight and normal-weight children. The means difference of FSIQ, VCI, WMI, PRI, and PSI among underweight and nonunderweight children were 4.06 (95% CI: 1.70–6.42), 3.55 (95% CI: 0.73–6.38), 3.19 (95% CI: 0.91–5.48), 4.41 (95% CI: 1.85–6.97), and 3.83 (95% CI: 1.35–6.31), respectively, after adjusting for confounders. The means of WISC-IV test scores were also lower in thin children. However, the associations were not significant before or after adjustment (Table 3). Similar associations were found between birth weight, HAZ, WAZ, and BAZ scores and WISC-IV test scores in repeated analyses after adding birth weight, HAZ, WAZ, and BAZ scores as continuous variables into the multilevel model.

## 4 Discussion

The present study shows that anthropometric indicators of low birth weight, stunting, and low body weight were negatively associated with the intellectual functioning of early school-aged children. Combined with the nonsignificant association between thinness and intellectual functioning, these results suggest that that early deficits in linear growth that are not recovered later in life and cumulative prenatal and postnatal malnutrition are significantly associated with intellectual impairment of early school-aged children.

The association between birth weight and childhood intelligence seems to be robust and similar to cultures with different socioeconomic history.^[23,24]^ Results from a meta-analysis study show that being born very low birth weight (<1500 g) is associated with significant motor impairment persisting throughout childhood.^[25]^ A longitudinal birth cohort study in England reported that low birth weight has a negative influence on cognitive function through to early adulthood.^[23]^ In the present study, we confirmed a consistent positive association between birth weight and childhood cognitive ability in rural western China, even when corrected for some confounders.

Postnatal nutrition status is also important for cognitive ability, it is well documented that suffering from malnutrition during the school years can inhibit a child's physical and mental development.^[6]^ In detail, results of a cross-sectional study in southeast Asian show that stunting, underweight, and thinness in the 4 countries (Indonesia, Malaysia, Thailand, and Vietnam) significantly increased the odds of children having a nonverbal IQ <89.^[26]^ Results from the Indian study show a negative association between underweight and stunting and cognitive ability.^[27]^ In addition, underweight and stunting are also reported associated with apathy, lower levels of play, and more insecure attachment than in nongrowth-retarded children.^[28,29]^ In the present study, the influence of stunting, low body weight on intellectual functioning at age 7 to 10 years was consistent with findings from previous studies. But the thinness was nonsignificantly associated with intellectual functioning in the present study. The sample in the southeast Asian cross-sectional study included children in urban areas, and children in present study were all from rural areas. Evidence suggests that higher levels of stimulation and learning opportunities are available to urban children as opposed to their rural counterparts.^[26]^ This may explain the effect of thinness on cognitive ability was different in 2 studies.

Previous studies have mostly reported impacts of malnutrition on FSIQ that represent overall cognitive ability or some specific dimensions of intellectual functioning, and few studies have systematically reported the effect of prenatal and postnatal malnutrition on intellectual functioning in early school-aged children. In the present study, the results show that stunting and low body weight were not the only causes of FSIQ impairments but also impairments to the other 4 dimensions of intellectual functioning. Low birth weight causes FSIQ and impairments in 3 other dimensions of intellectual functioning (all but WMI impairments) after adjusting for confounders. These observed negative effects of low birth weight, stunting, low body weight on intellectual functioning of school-aged children confirm the importance of prenatal and postnatal nutrition and, ideally, nutritional intervention should focus on pregnant women and children in early life. In addition, nutritional interventions targeting malnourished school-aged children should also be promoted to prevent the continuation of the stunting process.^[6]^

The effect of prenatal and postnatal malnutrition on the intellectual functioning of children is often caused by multiple factors, including micronutrient deficiencies, protein energy malnutrition, and chronic and recurrent infections.^[30]^ One possible explanation of the relationships between malnutrition and intellectual functioning is that undernourishment in children is associated with behavioral changes, which lead to poor developmental outcomes and poorer intellectual performance over the longer term. In addition, previous studies have reported that well nourished children were stimulated more than undernourished children by care givers, although it is unclear whether this is a reaction to the behaviors of undernourished children or precedes the development of malnutrition.^[9]^ Furthermore, the influences of low socioeconomic background on the malnutrition of children are well accepted, and many studies have also found that socioeconomic backgrounds such as poverty, low levels of maternal education, and decreased stimulation influence levels of stimulation, learning opportunities of children, and the quality of maternal–child interactions.^[9,19,31]^ The influences of maternal education, the household wealth index, and levels of schooling were also confirmed in the present study.

Our study has several strengths and some limitations. Because of the longitudinal nature of the data, we could reveal linkages between low birth weight and the intellectual functioning of children. Gestational information such as birth weight and gestational weeks at birth was collected at the time of birth, ensuring that this study does not suffer the potential biases that may be introduced when using recalled information. We used a well known, standardized measure of intellectual functioning available for use in different cultures and the latest version of WISC (WISC-IV) to test the intellectual functioning of 7 to 10-year-old children, which can better reveal the influences of malnutrition on different dimensions of cognitive development in children. A limitation of the present study was that we could not determine the mechanisms through which low birth weight, stunting, and low body weight reflect prenatal and postnatal malnutrition could independently affect the cognitive development of children. In addition, we did not include all possible confounders (such as the diet of the children or smoking) in our analysis, mainly because the relevance of these same dietary habits and the very small numbers of smokers among children. Other confounders (such as attention deficit hyperactivity disorder) were not excluded in the present study. Within the limitations, our results could also provide evidence for the effect of prenatal and postnatal malnutrition on intellectual function impairment.

## 5 Conclusion

In conclusion, our results suggest that prenatal (low birth weight) and postnatal (stunting and underweight) malnutrition influence the intellectual functioning of early school-aged children. The clear and strong associations between prenatal and postnatal malnutrition and intellectual functioning in the present study highlight the importance of establishing strategies to target nutritional concerns in pregnant women and school-aged children. Additional studies are required to determine the mechanisms by which prenatal and postnatal malnutrition influence the intellectual functioning of early school-aged children.

## Acknowledgments

Thanks to the National Natural Science Foundation of China for support (No: 81230016); the Health Departments of each project county, the local health bureaus, and education bureaus for their cooperation and organization of the field data collection; and the staff of Xi’an Jiaotong University for their participation in the field data collection.

## References

[1] Black RE Allen LH Bhutta ZA Maternal and child undernutrition: global and regional exposures and health consequences. *Lancet* 2008; 371:243–260. 18207566 10.1016/S0140-6736(07)61690-0

[2] Brown JL Pollitt E Malnutrition, poverty and intellectual development. *Sci Am* 1996; 274:38–43. 8560214 10.1038/scientificamerican0296-38

[3] Ivanovic R Forno H Castro CG Intellectual ability and nutritional status assessed through anthropometric measurements of Chilean school-age children from different socioeconomic status. *Ecol Food Nutr* 2000; 39:35–59.

[4] Bass BM Bass & Stogdill's Handbook of Leadership: Theory, Research, and Managerial Applications. 3rd ed. New York: Simon and Schuster; 1990.

[5] Grantham-McGregor S Cheung YB Cueto S Developmental potential in the first 5 years for children in developing countries. *Lancet* 2007; 369:60–70. 17208643 10.1016/S0140-6736(07)60032-4 PMC2270351

[6] Best C Neufingerl N van Geel L The nutritional status of school-aged children: why should we care? *Food Nutr Bull* 2010; 31:400–417. 20973461 10.1177/156482651003100303

[7] Morgane PJ Austin-La France R Bronzino J Prenatal malnutrition and development of the brain. *Neurosci Biobehav Rev* 1993; 17:91–128. 8455820 10.1016/s0149-7634(05)80234-9

[8] Lutter CK Chaparro CM Munoz S Progress towards Millennium Development Goal 1 in Latin America and the Caribbean: the importance of the choice of indicator for undernutrition. *Bull World Health Organ* 2011; 89:22–30. 21346887 10.2471/BLT.10.078618 PMC3040018

[9] WHO Expert Committee. Physical status: the use and interpretation of anthropometry. *World Health Organ Tech Rep Ser* 1995; 854:55. 8594834

[10] Lynn R What has caused the Flynn effect? Secular increases in the development quotients of infants. *Intelligence* 2009; 37:16–24.

[11] Shenkin SD Starr JM Deary IJ Birth weight and cognitive ability in childhood: a systematic review. *Psychol Bull* 2004; 130:989–1030. 15535745 10.1037/0033-2909.130.6.989

[12] Grantham-McGregor S Baker-Henningham H Review of the evidence linking protein and energy to mental development. *Pub Health Nutr* 2005; 8:1191–1201. 16277829 10.1079/phn2005805

[13] Victora CG Adair L Fall C Maternal and child undernutrition: consequences for adult health and humancapital. *Lancet* 2008; 3:340–357. 18206223 10.1016/S0140-6736(07)61692-4 PMC2258311

[14] Zeng L Dibley MJ Cheng Y Impact of micronutrient supplementation during pregnancy on birth weight, duration of gestation, and perinatal mortality in rural western China: double blind cluster randomised controlled trial. *BMJ* 2008; 337:a2001. 18996930 10.1136/bmj.a2001 PMC2577799

[15] Li Q Yan H Zeng L Effects of maternal multimicronutrient supplementation on the mental development of infants in rural western China: follow-up evaluation of a double-blind, randomized, controlled trial. *Pediatrics* 2009; 123:e685–e692. 19336358 10.1542/peds.2008-3007

[16] de Onis M Onyango AW Borghi E Development of a WHO growth reference for school-aged children and adolescents. *Bull World Health Organ* 2007; 85:660–667. 18026621 10.2471/BLT.07.043497 PMC2636412

[17] Wechsler D Manual for the Wechsler Intelligence Scale for Children. 4th ed. San Antonio: The Psychological Corporation; 2003.

[18] Chen H Keith TZ Weiss L Testing for multigroup invariance of second-order WISC-IV structure across China, Hong Kong, Macau, and Taiwan. *Pers Individ Differences* 2010; 49:677–682.

[19] Walker SP Wachs TD Gardner JM Child development: risk factors for adverse outcomes in developing countries. *Lancet* 2007; 369:149–157. 10.1016/S0140-6736(07)60076-2 17223478

[20] Li C Zeng L Wang D Prenatal micronutrient supplementation is not associated with intellectual development of young school-aged children. *J Nutr* 2015; 145:1844–1849. 26084366 10.3945/jn.114.207795

[21] Li C Zhu N Zeng L Sex differences in the intellectual functioning of early school-aged children in rural China. *BMC Public Health* 2016; 16:1. 27026407 10.1186/s12889-016-2956-6 PMC4812622

[22] Christian P Murray-Kolb LE Khatry SK Prenatal micronutrient supplementation and intellectual and motor function in early school-aged children in Nepal. *JAMA* 2010; 304:2716–2723. 21177506 10.1001/jama.2010.1861

[23] Jefferis BJ Power C Hertzman C Birth weight, childhood socioeconomic environment, and cognitive development in the 1958 British birth cohort study. *BMJ* 2002; 325:305. 12169505 10.1136/bmj.325.7359.305 PMC117769

[24] Richards M Hardy R Kuh D Birthweight, postnatal growth and cognitive function in a national UK birth cohort. *Int J Epidemiol* 2002; 31:342–348. 11980795

[25] de Kieviet JF Zoetebier L Van Elburg RM Brain development of very preterm and very low-birthweight children in childhood and adolescence: a meta-analysis. *Dev Med Child Neurol* 2012; 54:313–323. 22283622 10.1111/j.1469-8749.2011.04216.x

[26] Sandjaja Poh BK Rojroonwasinkul N Relationship between anthropometric indicators and cognitive performance in Southeast Asian school-aged children. *Br J Nutr* 2013; 110 suppl 3:S57–S64. 24016767 10.1017/S0007114513002079

[27] Malhi P Bharti B Sidhu M Developmental functioning of young Indian children with malnutrition. *Psychol Stud* 2013; 58:259–264.

[28] Casale D Desmond C Richter L The association between stunting and psychosocial development among preschool children: a study using the South African birth to twenty cohort data. *Child Care Health Dev* 2014; 40:900–910. 24807234 10.1111/cch.12143

[29] Eilander A Muthayya S van der Knaap Undernutrition, fatty acid and micronutrient status in relation to cognitive performance in Indian school children: a cross sectional study. *Br J Nutr* 2010; 103:1056–1064. 20003612 10.1017/S000711450999273X

[30] Rosenberg M Global child health: burden of disease, achievements and future challenges. *Curr Probl Pediatr Adolesc Health Care* 2007; 37:338–362. 17916531 10.1016/j.cppeds.2007.07.003

[31] United Nations Children's Fund. Tracking Progress on Child and Maternal Nutrition: A Survival and Development Priority. New York, NY: UNICEF; 2009.

